# Hyperinflammation due to COVID-19 and the Targeted Use of Interleukin-1 Inhibitors

**DOI:** 10.31138/mjr.33.2.173

**Published:** 2022-06-30

**Authors:** Armen Yuri Gasparyan, George D. Kitas

**Affiliations:** 1 Departments of Rheumatology and Research and Development, Dudley Group NHS Foundation Trust (Teaching Trust of the University of Birmingham, UK), Russells Hall Hospital, Dudley, West Midlands, United Kingdom,; 2 Centre for Epidemiology versus Arthritis, University of Manchester, Manchester, United Kingdom

**Keywords:** anakinra, canakinumab, COVID-19, cytokine release syndrome, hereditary autoinflammatory diseases, interleukin-1, interleukin-6

The COVID-19 pandemic has renewed the interest towards infectious agents breaching human immune defence systems, triggering (auto)antibody production and resulting in (auto)immune inflammatory manifestations. The initial months of the pandemic were marked by the description of COVID-19 systemic manifestations, severity prediction, and an empirical treatment approach to the infection. One of the dreadful manifestations of COVID-19, Acute Respiratory Distress Syndrome (ARDS), was described as cytokine storm due to immune dysregulation, hyperinflammation, and overproduction of inflammatory cytokines.^1^ It was hypothesised that the usually protective production of inflammatory cytokines at a certain quantitative threshold could turn the tide against human organ systems and result in severe COVID-19, necessitating anti-cytokine therapies.^2^ The overproduction of IL-1beta, Interleukin (IL)-6, and Tumour Necrosis Factor (TNF)-alpha was often described in severe COVID-19.^3^ Monocyte dysregulation with uncontrolled IL-6 production was found to be the most powerful link in the immune and inflammatory cascade in severe COVID-19.^4^ Activation of inflammasomes by infected macrophages was described as another pathogenic link in severe COVID-19, releasing IL-1 and IL-18 and contributing to the hyperinflammation in the lungs.^5^ Hyperinflammation with a predominant action of IL-6 is known as a pathogenic factor of COVID-19-related arthritis that affects ankles, knees, and wrists in a sizable proportion of patients (37%).^6^ Several large cohort studies have shown no difference in the risk of severe COVID-19 and related mortality between patients with inflammatory rheumatic diseases (IRD) and the general population.^7–9^ Patients with IRD at old age, those with comorbidities such as arterial hypertension and neoplasms, and those on corticosteroid and rituximab therapies were found at increased risk of severe COVID-19, hospitalisation, and intensive care unit submission.^10,11^

With improved understanding of cellular pathways and the interrelated IL-1 and IL-6 pathogenic roles in COVID-19, several anti-cytokine therapies were proposed in the early months of the pandemic.^12^ Both anti-IL-1 and anti-IL-6 therapies were tested as life-saving options in severe COVID-19 with resultant discrepancies in the outcomes of the randomized controlled trials. An initial randomised, double-blind, placebo-controlled trial involving moderately-ill hospitalised patients with COVID-19 (n=243) on supplemental oxygen failed to reveal any effect of anti-IL-6 therapy with 8 mg/kg tocilizumab on preventing intubation or death within 2 weeks.^13^

However, another trial of 90-day survival benefit in critically-ill COVID-19 patients supported the use of anti-IL-6 therapies with tocilizumab and sarilumab.^14^ Also, the life-saving benefit of tocilizumab within 4 weeks was demonstrated in a randomised, controlled, open-label, platform trial (Randomised Evaluation of COVID-19 Therapy [RECOVERY]; n=4116) where 82% of patients received systemic corticosteroids.^15^

Importantly, early inhibition of inflammation with the IL-1 antagonist, anakinra (within 36 hours of ARDS) was tested to prevent mechanical ventilation in COVID-19.^16^ A phase 3 trial of anakinra in COVID-19 patients who were predominantly treated with dexamethasone revealed a survival benefit at 28 days with borderline significance (Hazard Ratio 0.45, P=0.045).^17^ The beneficial effect of anakinra plus methylprednisolone at 28 days was also demonstrated in a prospective cohort study of COVID-19 patients with or without mechanical ventilation (n=120).^18^ However, a trial of subcutaneous administration of anakinra in patients with moderate-to-severe COVID-19 pneumonia requiring supplementary oxygen failed to demonstrate any survival benefit at 90 days.^19^ Preliminary evidence from scarce studies of patients with autoinflammatory diseases exposed to COVID-19 further improves our understanding of the predictive role of IL-1 inhibition in these clinical models of chronic inflammation. Initially, familial Mediterranean fever (FMF), the prototype autoinflammatory disease with overactivation of the pyrin inflammasome, was even considered an entity overlapping with COVID-19 in a number of clinical manifestations, C-reactive protein overproduction, leucocytosis, and mild cytokine storm.^20^ A Turkish nationwide cross-sectional study of FMF patients contracting COVID-19 (n=59) did not report any lethal outcome.^21^ All the examined patients were on regular colchicine, 1 on anakinra, and 1 on canakinumab therapies. In this study, hospitalised patients were significantly older than non-hospitalised patients (median age 51 vs 31, P=0.002).^21^ In a small group of patients with FMF contracting COVID-19 those treated with anakinra (n=8) due to poorly controlled systemic inflammation were often hospitalized (n=4).^22^

The results of a French prospective follow-up of patients with systemic autoinflammatory diseases such as FMF and Behçet disease (BD) did not reveal any risk of severe COVID-19 in those treated with anakinra therapy.^23^ Other factors such as age, comorbidities, and chronic corticosteroid therapies were discussed in connection with the risk of severe infection.^23^

Overall, there is lack of robust evidence to support the use of IL-1 inhibitors for severe COVID-19 in the general population and in patients with autoinflammatory diseases. The latest Cochrane systematic review has summarised data from four completed trials of IL-1 inhibitors in COVID-19 without any encouraging results due to the safety and efficacy uncertainties.^24^ Consequently, the question arises whether patients with autoinflammatory diseases with suppressed IL-1 activity and preserved B lymphocytes are on a safe side in the context of severe COVID-19. Theoretically, the exposure to the novel coronavirus may lead to asymptomatic or mild infection in these patients. Also, the preserved activity of plasmocytes in autoinflammatory diseases may result in sufficient production of neutralizing antibodies in response to COVID-19 vaccination.^25^ Nonetheless, there are still no specific guidelines on the use of IL-1 inhibitors in patients with autoinflammatory diseases and COVID-19 or those planning COVID-19 vaccination. What is certain in the COVID-19 pandemic is that caution should be exercised in patients at advanced age, those with comorbidities, and those on chronic corticosteroid and/or B-cell-depleting therapies.
